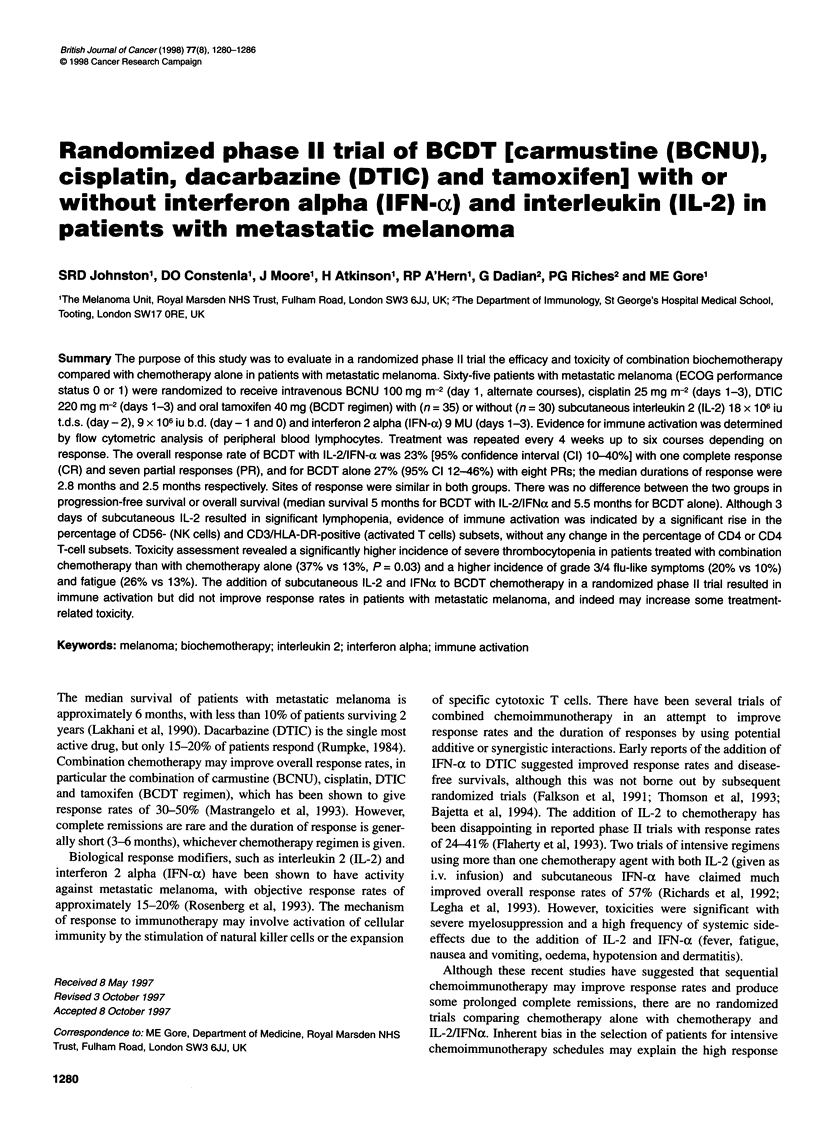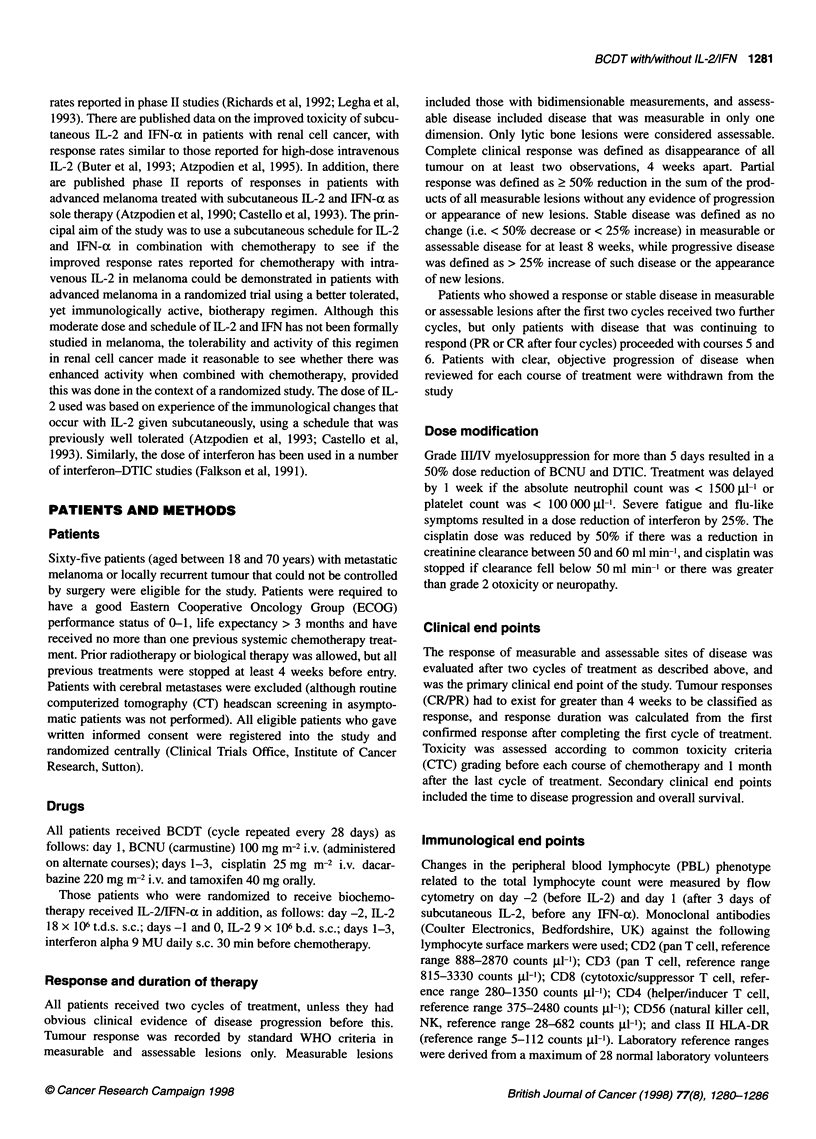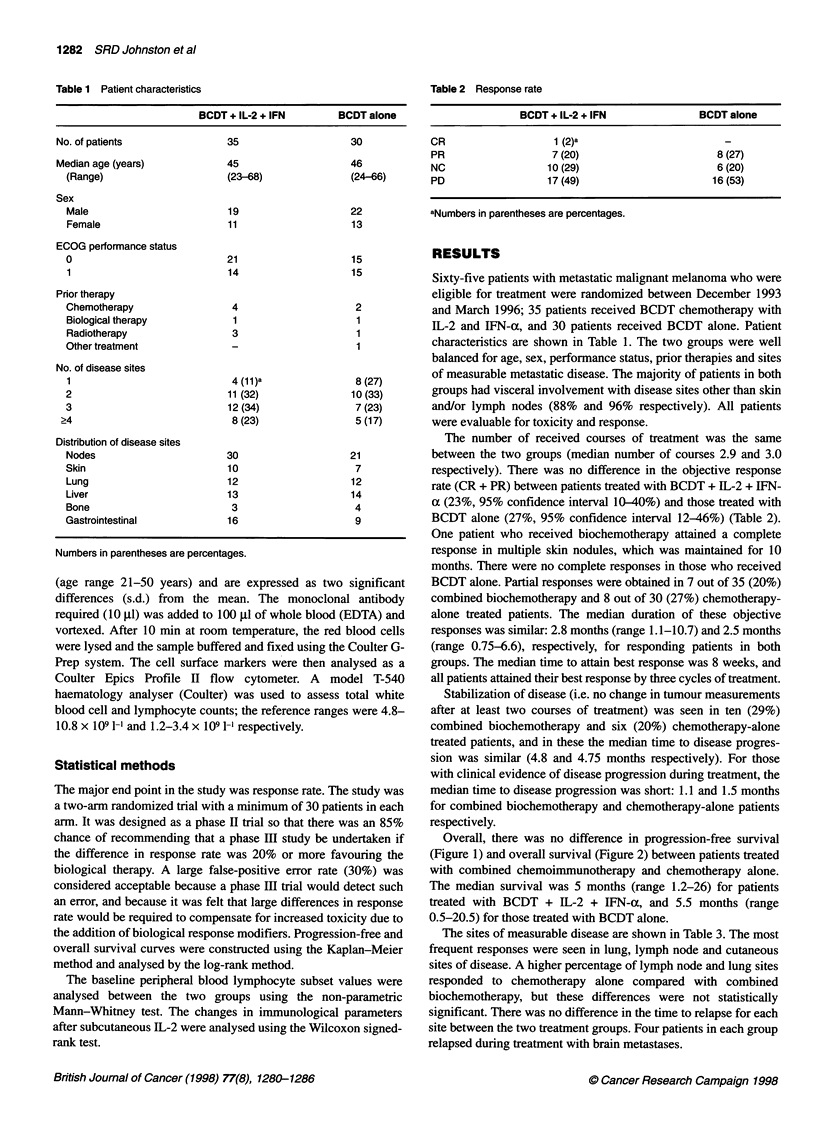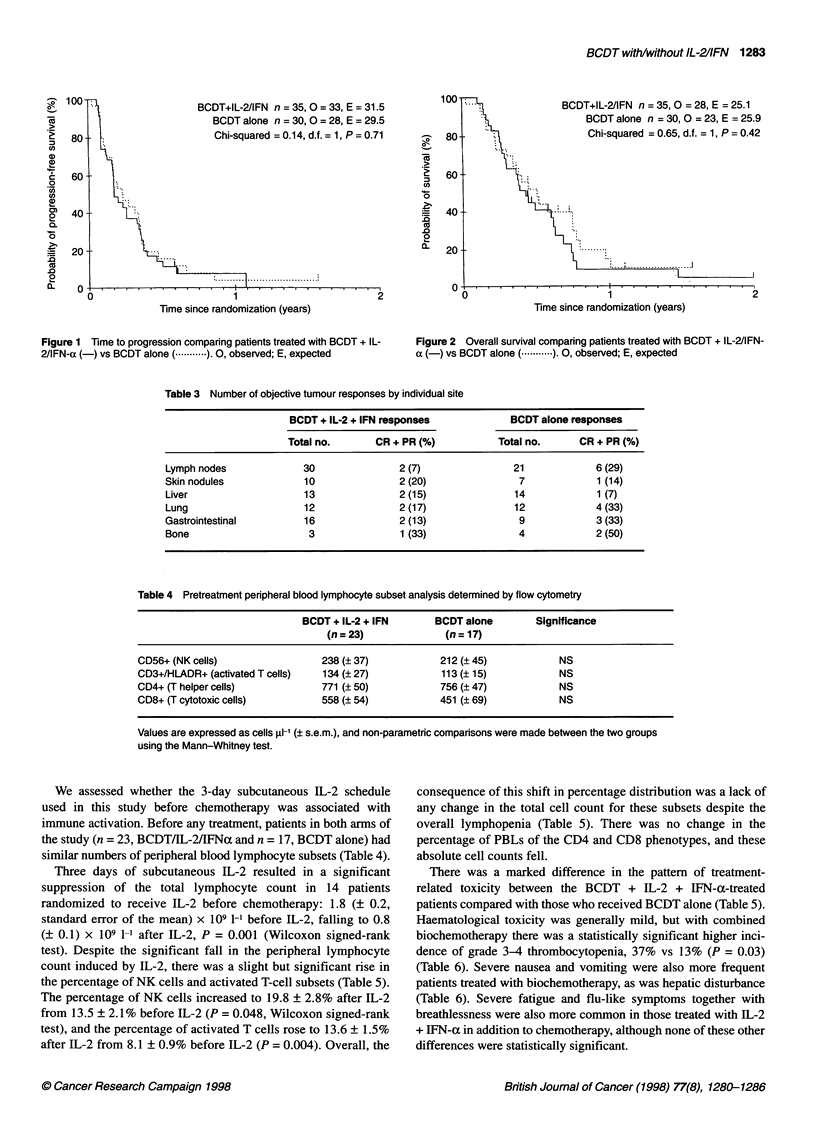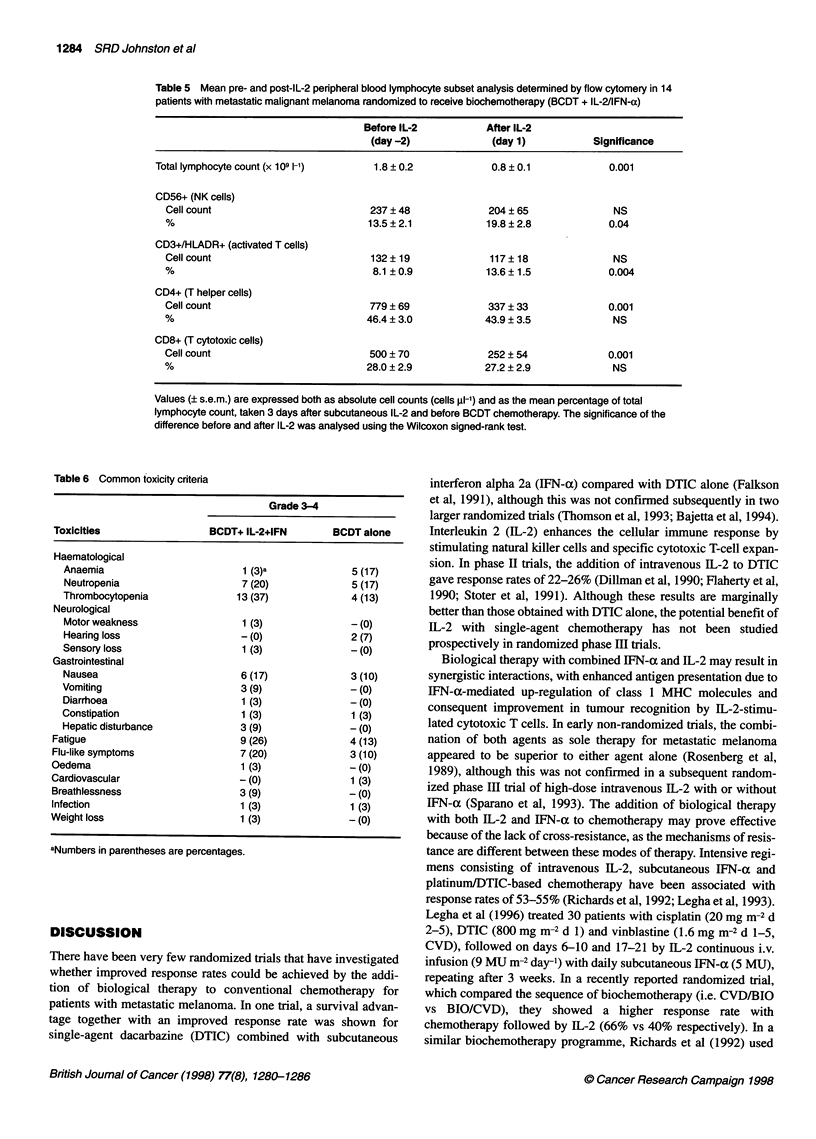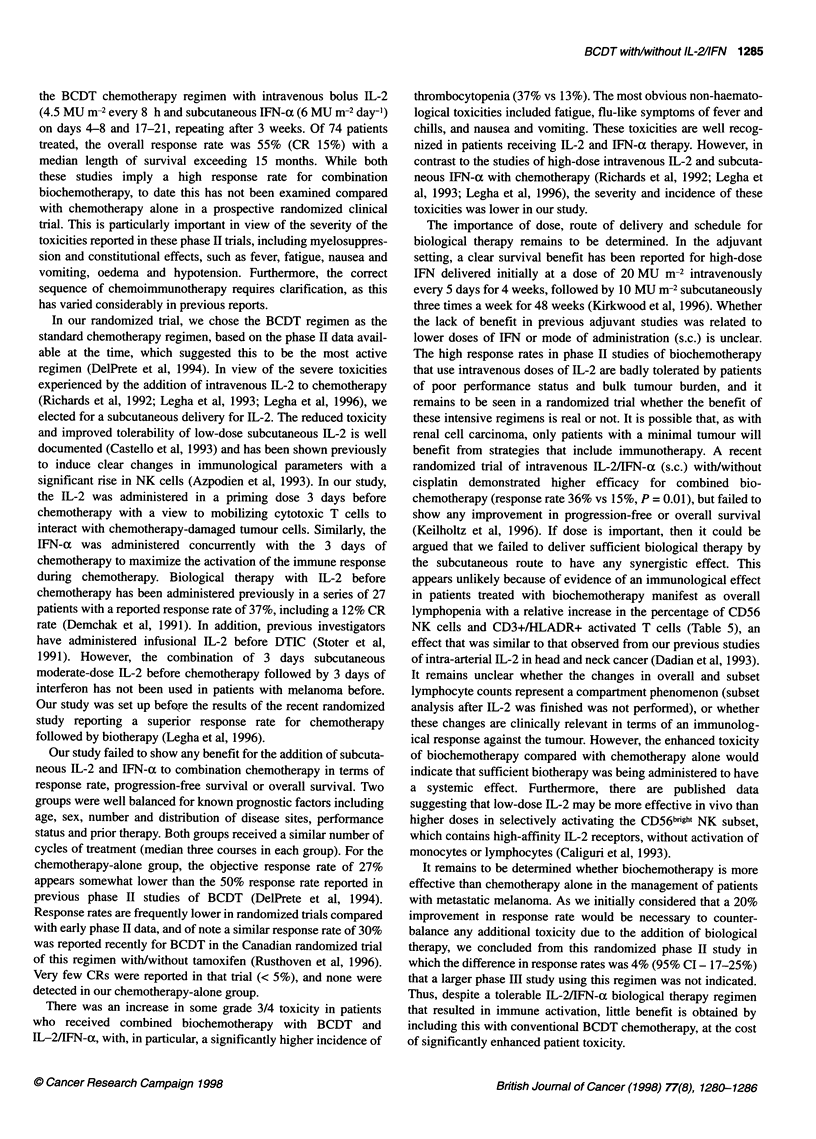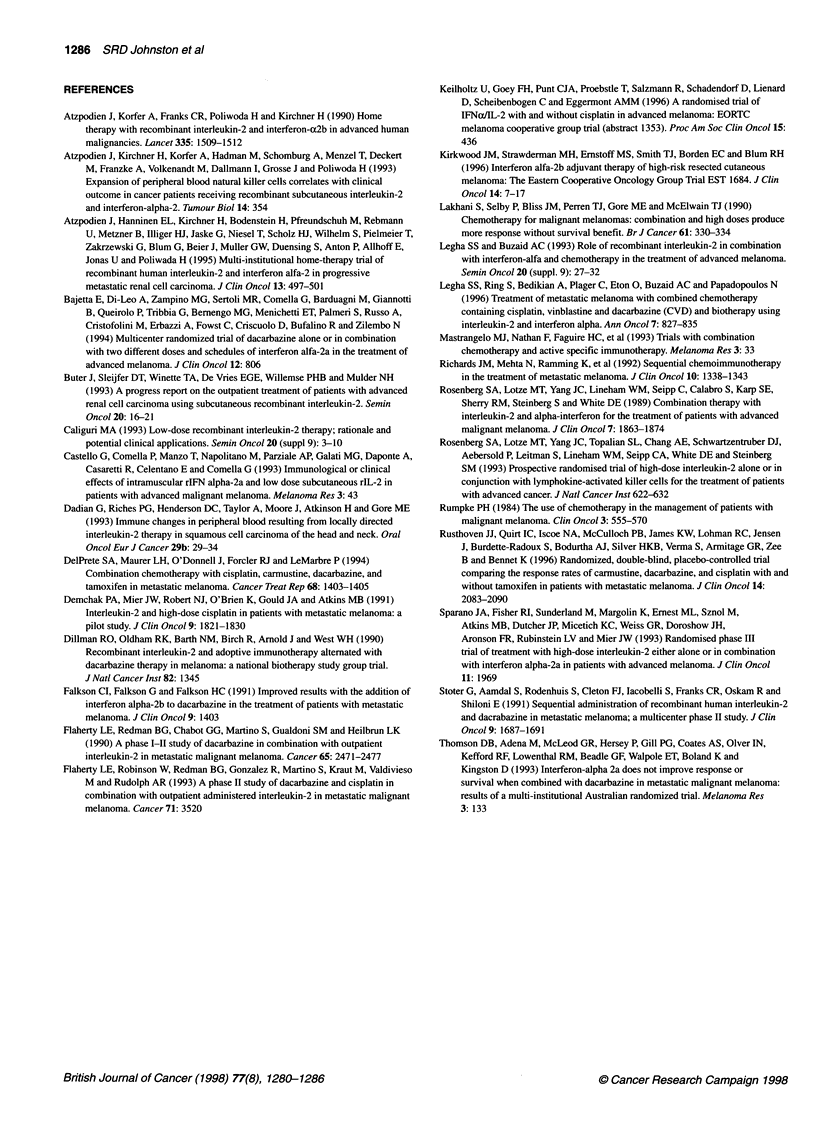# Randomized phase II trial of BCDT [carmustine (BCNU), cisplatin, dacarbazine (DTIC) and tamoxifen] with or without interferon alpha (IFN-alpha) and interleukin (IL-2) in patients with metastatic melanoma.

**DOI:** 10.1038/bjc.1998.214

**Published:** 1998-04

**Authors:** S. R. Johnston, D. O. Constenla, J. Moore, H. Atkinson, R. P. A'Hern, G. Dadian, P. G. Riches, M. E. Gore

**Affiliations:** The Melanoma Unit, Royal Marsden NHS Trust, London, UK.

## Abstract

The purpose of this study was to evaluate in a randomized phase II trial the efficacy and toxicity of combination biochemotherapy compared with chemotherapy alone in patients with metastatic melanoma. Sixty-five patients with metastatic melanoma (ECOG performance status 0 or 1) were randomized to receive intravenous BCNU 100 mg m(-2) (day 1, alternate courses), cisplatin 25 mg m(-2) (days 1-3), DTIC 220 mg m(-2) (days 1-3) and oral tamoxifen 40 mg (BCDT regimen) with (n = 35) or without (n = 30) subcutaneous interleukin 2 (IL-2) 18 x 10(6) iu t.d.s. (day - 2), 9 x 10(6) iu b.d. (day - 1 and 0) and interferon 2 alpha (IFN-alpha) 9 MU (days 1-3). Evidence for immune activation was determined by flow cytometric analysis of peripheral blood lymphocytes. Treatment was repeated every 4 weeks up to six courses depending on response. The overall response rate of BCDT with IL-2/IFN-alpha was 23% [95% confidence interval (CI) 10-40%] with one complete response (CR) and seven partial responses (PR), and for BCDT alone 27% (95% CI 12-46%) with eight PRs; the median durations of response were 2.8 months and 2.5 months respectively. Sites of response were similar in both groups. There was no difference between the two groups in progression-free survival or overall survival (median survival 5 months for BCDT with IL-2/IFNalpha and 5.5 months for BCDT alone). Although 3 days of subcutaneous IL-2 resulted in significant lymphopenia, evidence of immune activation was indicated by a significant rise in the percentage of CD56- (NK cells) and CD3/HLA-DR-positive (activated T cells) subsets, without any change in the percentage of CD4 or CD4 T-cell subsets. Toxicity assessment revealed a significantly higher incidence of severe thrombocytopenia in patients treated with combination chemotherapy than with chemotherapy alone (37% vs 13%, P = 0.03) and a higher incidence of grade 3/4 flu-like symptoms (20% vs 10%) and fatigue (26% vs 13%). The addition of subcutaneous IL-2 and IFNalpha to BCDT chemotherapy in a randomized phase II trial resulted in immune activation but did not improve response rates in patients with metastatic melanoma, and indeed may increase some treatment-related toxicity.


*British Journal of Cancer* (1998) **77**(8), 1280–1286
© 1998 Cancer Research Campaign

### Randomized phase II trial of BCDT [carmustine (BCNU), cisplatin, dacarbazine (DTIC) and tamoxifen] with or without interferon alpha (IFN-α) and interleukin (IL-2) in patients with metastatic melanoma

**SRD Johnston[1], DO Constenla[1], J Moore[1], H Atkinson[1], RP A'Hern[1], G Dadian[2], PG Riches[2] and ME Gore[1]**

[1]The Melanoma Unit, Royal Marsden NHS Trust, Fulham Road, London SW3 6JJ, UK; [2]The Department of Immunology, St George's Hospital Medical School, Tooting, London SW17 0RE, UK

**Summary** The purpose of this study was to evaluate in a randomized phase II trial the efficacy and toxicity of combination biochemotherapy compared with chemotherapy alone in patients with metastatic melanoma. Sixty-five patients with metastatic melanoma (ECOG performance status 0 or 1) were randomized to receive intravenous BCNU 100 mg $m^{-2}$ (day 1, alternate courses), cisplatin 25 mg $m^{-2}$ (days 1–3), DTIC 220 mg $m^{-2}$ (days 1–3) and oral tamoxifen 40 mg (BCDT regimen) with ($n = 35$) or without ($n = 30$) subcutaneous interleukin 2 (IL-2) $18 \times 10^6$ iu t.d.s. (day – 2), $9 \times 10^6$ iu b.d. (day – 1 and 0) and interferon 2 alpha (IFN-α) 9 MU (days 1–3). Evidence for immune activation was determined by flow cytometric analysis of peripheral blood lymphocytes. Treatment was repeated every 4 weeks up to six courses depending on response. The overall response rate of BCDT with IL-2/IFN-α was 23% [95% confidence interval (CI) 10–40%] with one complete response (CR) and seven partial responses (PR), and for BCDT alone 27% (95% CI 12–46%) with eight PRs; the median durations of response were 2.8 months and 2.5 months respectively. Sites of response were similar in both groups. There was no difference between the two groups in progression-free survival or overall survival (median survival 5 months for BCDT with IL-2/IFNα and 5.5 months for BCDT alone). Although 3 days of subcutaneous IL-2 resulted in significant lymphopenia, evidence of immune activation was indicated by a significant rise in the percentage of CD56- (NK cells) and CD3/HLA-DR-positive (activated T cells) subsets, without any change in the percentage of CD4 or CD4 T-cell subsets. Toxicity assessment revealed a significantly higher incidence of severe thrombocytopenia in patients treated with combination chemotherapy than with chemotherapy alone (37% vs 13%, $P = 0.03$) and a higher incidence of grade 3/4 flu-like symptoms (20% vs 10%) and fatigue (26% vs 13%). The addition of subcutaneous IL-2 and IFNα to BCDT chemotherapy in a randomized phase II trial resulted in immune activation but did not improve response rates in patients with metastatic melanoma, and indeed may increase some treatment-related toxicity.

**Keywords:** melanoma; biochemotherapy; interleukin 2; interferon alpha; immune activation

The median survival of patients with metastatic melanoma is approximately 6 months, with less than 10% of patients surviving 2 years (Lakhani et al, 1990). Dacarbazine (DTIC) is the single most active drug, but only 15–20% of patients respond (Rumpke, 1984). Combination chemotherapy may improve overall response rates, in particular the combination of carmustine (BCNU), cisplatin, DTIC and tamoxifen (BCDT regimen), which has been shown to give response rates of 30–50% (Mastrangelo et al, 1993). However, complete remissions are rare and the duration of response is generally short (3–6 months), whichever chemotherapy regimen is given.

Biological response modifiers, such as interleukin 2 (IL-2) and interferon 2 alpha (IFN-α) have been shown to have activity against metastatic melanoma, with objective response rates of approximately 15–20% (Rosenberg et al, 1993). The mechanism of response to immunotherapy may involve activation of cellular immunity by the stimulation of natural killer cells or the expansion of specific cytotoxic T cells. There have been several trials of combined chemoimmunotherapy in an attempt to improve response rates and the duration of responses by using potential additive or synergistic interactions. Early reports of the addition of IFN-α to DTIC suggested improved response rates and disease-free survivals, although this was not borne out by subsequent randomized trials (Falkson et al, 1991; Thomson et al, 1993; Bajetta et al, 1994). The addition of IL-2 to chemotherapy has been disappointing in reported phase II trials with response rates of 24–41% (Flaherty et al, 1993). Two trials of intensive regimens using more than one chemotherapy agent with both IL-2 (given as i.v. infusion) and subcutaneous IFN-α have claimed much improved overall response rates of 57% (Richards et al, 1992; Legha et al, 1993). However, toxicities were significant with severe myelosuppression and a high frequency of systemic side-effects due to the addition of IL-2 and IFN-α (fever, fatigue, nausea and vomiting, oedema, hypotension and dermatitis).

Although these recent studies have suggested that sequential chemoimmunotherapy may improve response rates and produce some prolonged complete remissions, there are no randomized trials comparing chemotherapy alone with chemotherapy and IL-2/IFNα. Inherent bias in the selection of patients for intensive chemoimmunotherapy schedules may explain the high response

*Received 8 May 1997*
*Revised 3 October 1997*
*Accepted 8 October 1997*

*Correspondence to:* ME Gore, Department of Medicine, Royal Marsden NHS Trust, Fulham Road, London SW3 6JJ, UK

rates reported in phase II studies (Richards et al, 1992; Legha et al, 1993). There are published data on the improved toxicity of subcutaneous IL-2 and IFN-α in patients with renal cell cancer, with response rates similar to those reported for high-dose intravenous IL-2 (Buter et al, 1993; Atzpodien et al, 1995). In addition, there are published phase II reports of responses in patients with advanced melanoma treated with subcutaneous IL-2 and IFN-α as sole therapy (Atzpodien et al, 1990; Castello et al, 1993). The principal aim of the study was to use a subcutaneous schedule for IL-2 and IFN-α in combination with chemotherapy to see if the improved response rates reported for chemotherapy with intravenous IL-2 in melanoma could be demonstrated in patients with advanced melanoma in a randomized trial using a better tolerated, yet immunologically active, biotherapy regimen. Although this moderate dose and schedule of IL-2 and IFN has not been formally studied in melanoma, the tolerability and activity of this regimen in renal cell cancer made it reasonable to see whether there was enhanced activity when combined with chemotherapy, provided this was done in the context of a randomized study. The dose of IL-2 used was based on experience of the immunological changes that occur with IL-2 given subcutaneously, using a schedule that was previously well tolerated (Atzpodien et al, 1993; Castello et al, 1993). Similarly, the dose of interferon has been used in a number of interferon–DTIC studies (Falkson et al, 1991).

### PATIENTS AND METHODS

### Patients

Sixty-five patients (aged between 18 and 70 years) with metastatic melanoma or locally recurrent tumour that could not be controlled by surgery were eligible for the study. Patients were required to have a good Eastern Cooperative Oncology Group (ECOG) performance status of 0–1, life expectancy > 3 months and have received no more than one previous systemic chemotherapy treatment. Prior radiotherapy or biological therapy was allowed, but all previous treatments were stopped at least 4 weeks before entry. Patients with cerebral metastases were excluded (although routine computerized tomography (CT) headscan screening in asymptomatic patients was not performed). All eligible patients who gave written informed consent were registered into the study and randomized centrally (Clinical Trials Office, Institute of Cancer Research, Sutton).

### Drugs

All patients received BCDT (cycle repeated every 28 days) as follows: day 1, BCNU (carmustine) 100 mg $m^{-2}$ i.v. (administered on alternate courses); days 1–3, cisplatin 25 mg $m^{-2}$ i.v. dacarbazine 220 mg $m^{-2}$ i.v. and tamoxifen 40 mg orally.

Those patients who were randomized to receive biochemotherapy received IL-2/IFN-α in addition, as follows: day –2, IL-2 $18 \times 10^6$ t.d.s. s.c.; days –1 and 0, IL-2 $9 \times 10^6$ b.d. s.c.; days 1–3, interferon alpha 9 MU daily s.c. 30 min before chemotherapy.

### Response and duration of therapy

All patients received two cycles of treatment, unless they had obvious clinical evidence of disease progression before this. Tumour response was recorded by standard WHO criteria in measurable and assessable lesions only. Measurable lesions included those with bidimensionable measurements, and assessable disease included disease that was measurable in only one dimension. Only lytic bone lesions were considered assessable. Complete clinical response was defined as disappearance of all tumour on at least two observations, 4 weeks apart. Partial response was defined as ≥ 50% reduction in the sum of the products of all measurable lesions without any evidence of progression or appearance of new lesions. Stable disease was defined as no change (i.e. < 50% decrease or < 25% increase) in measurable or assessable disease for at least 8 weeks, while progressive disease was defined as > 25% increase of such disease or the appearance of new lesions.

Patients who showed a response or stable disease in measurable or assessable lesions after the first two cycles received two further cycles, but only patients with disease that was continuing to respond (PR or CR after four cycles) proceeded with courses 5 and 6. Patients with clear, objective progression of disease when reviewed for each course of treatment were withdrawn from the study

### Dose modification

Grade III/IV myelosuppression for more than 5 days resulted in a 50% dose reduction of BCNU and DTIC. Treatment was delayed by 1 week if the absolute neutrophil count was < 1500 $\mu l^{-1}$ or platelet count was < 100 000 $\mu l^{-1}$. Severe fatigue and flu-like symptoms resulted in a dose reduction of interferon by 25%. The cisplatin dose was reduced by 50% if there was a reduction in creatinine clearance between 50 and 60 ml $min^{-1}$, and cisplatin was stopped if clearance fell below 50 ml $min^{-1}$ or there was greater than grade 2 otoxicity or neuropathy.

### Clinical end points

The response of measurable and assessable sites of disease was evaluated after two cycles of treatment as described above, and was the primary clinical end point of the study. Tumour responses (CR/PR) had to exist for greater than 4 weeks to be classified as response, and response duration was calculated from the first confirmed response after completing the first cycle of treatment. Toxicity was assessed according to common toxicity criteria (CTC) grading before each course of chemotherapy and 1 month after the last cycle of treatment. Secondary clinical end points included the time to disease progression and overall survival.

### Immunological end points

Changes in the peripheral blood lymphocyte (PBL) phenotype related to the total lymphocyte count were measured by flow cytometry on day –2 (before IL-2) and day 1 (after 3 days of subcutaneous IL-2, before any IFN-α). Monoclonal antibodies (Coulter Electronics, Bedfordshire, UK) against the following lymphocyte surface markers were used; CD2 (pan T cell, reference range 888–2870 counts $\mu l^{-1}$); CD3 (pan T cell, reference range 815–3330 counts $\mu l^{-1}$); CD8 (cytotoxic/suppressor T cell, reference range 280–1350 counts $\mu l^{-1}$); CD4 (helper/inducer T cell, reference range 375–2480 counts $\mu l^{-1}$); CD56 (natural killer cell, NK, reference range 28–682 counts $\mu l^{-1}$); and class II HLA-DR (reference range 5–112 counts $\mu l^{-1}$). Laboratory reference ranges were derived from a maximum of 28 normal laboratory volunteers

© Cancer Research Campaign 1998 

**Table 1** Patient characteristics

| | BCDT + IL-2 + IFN | BCDT alone |
|---|---|---|
| No. of patients | 35 | 30 |
| Median age (years) | 45 | 46 |
| (Range) | (23–68) | (24–66) |
| Sex | | |
| Male | 19 | 22 |
| Female | 11 | 13 |
| ECOG performance status | | |
| 0 | 21 | 15 |
| 1 | 14 | 15 |
| Prior therapy | | |
| Chemotherapy | 4 | 2 |
| Biological therapy | 1 | 1 |
| Radiotherapy | 3 | 1 |
| Other treatment | – | 1 |
| No. of disease sites | | |
| 1 | 4 (11)[a] | 8 (27) |
| 2 | 11 (32) | 10 (33) |
| 3 | 12 (34) | 7 (23) |
| ≥4 | 8 (23) | 5 (17) |
| Distribution of disease sites | | |
| Nodes | 30 | 21 |
| Skin | 10 | 7 |
| Lung | 12 | 12 |
| Liver | 13 | 14 |
| Bone | 3 | 4 |
| Gastrointestinal | 16 | 9 |

Numbers in parentheses are percentages.

(age range 21–50 years) and are expressed as two significant differences (s.d.) from the mean. The monoclonal antibody required (10 µl) was added to 100 µl of whole blood (EDTA) and vortexed. After 10 min at room temperature, the red blood cells were lysed and the sample buffered and fixed using the Coulter G-Prep system. The cell surface markers were then analysed as a Coulter Epics Profile II flow cytometer. A model T-540 haematology analyser (Coulter) was used to assess total white blood cell and lymphocyte counts; the reference ranges were 4.8–10.8 $\times 10^9$ $l^{-1}$ and 1.2–3.4 $\times 10^9$ $l^{-1}$ respectively.

### Statistical methods

The major end point in the study was response rate. The study was a two-arm randomized trial with a minimum of 30 patients in each arm. It was designed as a phase II trial so that there was an 85% chance of recommending that a phase III study be undertaken if the difference in response rate was 20% or more favouring the biological therapy. A large false-positive error rate (30%) was considered acceptable because a phase III trial would detect such an error, and because it was felt that large differences in response rate would be required to compensate for increased toxicity due to the addition of biological response modifiers. Progression-free and overall survival curves were constructed using the Kaplan–Meier method and analysed by the log-rank method.

The baseline peripheral blood lymphocyte subset values were analysed between the two groups using the non-parametric Mann–Whitney test. The changes in immunological parameters after subcutaneous IL-2 were analysed using the Wilcoxon signed-rank test.

**Table 2** Response rate

| | BCDT + IL-2 + IFN | BCDT alone |
|---|---|---|
| CR | 1 (2)[a] | – |
| PR | 7 (20) | 8 (27) |
| NC | 10 (29) | 6 (20) |
| PD | 17 (49) | 16 (53) |

[a]Numbers in parentheses are percentages.

### RESULTS

Sixty-five patients with metastatic malignant melanoma who were eligible for treatment were randomized between December 1993 and March 1996; 35 patients received BCDT chemotherapy with IL-2 and IFN-α, and 30 patients received BCDT alone. Patient characteristics are shown in Table 1. The two groups were well balanced for age, sex, performance status, prior therapies and sites of measurable metastatic disease. The majority of patients in both groups had visceral involvement with disease sites other than skin and/or lymph nodes (88% and 96% respectively). All patients were evaluable for toxicity and response.

The number of received courses of treatment was the same between the two groups (median number of courses 2.9 and 3.0 respectively). There was no difference in the objective response rate (CR + PR) between patients treated with BCDT + IL-2 + IFN-α (23%, 95% confidence interval 10–40%) and those treated with BCDT alone (27%, 95% confidence interval 12–46%) (Table 2). One patient who received biochemotherapy attained a complete response in multiple skin nodules, which was maintained for 10 months. There were no complete responses in those who received BCDT alone. Partial responses were obtained in 7 out of 35 (20%) combined biochemotherapy and 8 out of 30 (27%) chemotherapy-alone treated patients. The median duration of these objective responses was similar: 2.8 months (range 1.1–10.7) and 2.5 months (range 0.75–6.6), respectively, for responding patients in both groups. The median time to attain best response was 8 weeks, and all patients attained their best response by three cycles of treatment.

Stabilization of disease (i.e. no change in tumour measurements after at least two courses of treatment) was seen in ten (29%) combined biochemotherapy and six (20%) chemotherapy-alone treated patients, and in these the median time to disease progression was similar (4.8 and 4.75 months respectively). For those with clinical evidence of disease progression during treatment, the median time to disease progression was short: 1.1 and 1.5 months for combined biochemotherapy and chemotherapy-alone patients respectively.

Overall, there was no difference in progression-free survival (Figure 1) and overall survival (Figure 2) between patients treated with combined chemoimmunotherapy and chemotherapy alone. The median survival was 5 months (range 1.2–26) for patients treated with BCDT + IL-2 + IFN-α, and 5.5 months (range 0.5–20.5) for those treated with BCDT alone.

The sites of measurable disease are shown in Table 3. The most frequent responses were seen in lung, lymph node and cutaneous sites of disease. A higher percentage of lymph node and lung sites responded to chemotherapy alone compared with combined biochemotherapy, but these differences were not statistically significant. There was no difference in the time to relapse for each site between the two treatment groups. Four patients in each group relapsed during treatment with brain metastases.

*© Cancer Research Campaign 1998*

**Figure 1** Time to progression comparing patients treated with BCDT + IL-2/IFN-α (—) vs BCDT alone (··········). O, observed; E, expected

**Figure 2** Overall survival comparing patients treated with BCDT + IL-2/IFN-α (—) vs BCDT alone (··········). O, observed; E, expected

**Table 3** Number of objective tumour responses by individual site

| | BCDT + IL-2 + IFN responses | | BCDT alone responses | |
|---|---|---|---|---|
| | Total no. | CR + PR (%) | Total no. | CR + PR (%) |
| Lymph nodes | 30 | 2 (7) | 21 | 6 (29) |
| Skin nodules | 10 | 2 (20) | 7 | 1 (14) |
| Liver | 13 | 2 (15) | 14 | 1 (7) |
| Lung | 12 | 2 (17) | 12 | 4 (33) |
| Gastrointestinal | 16 | 2 (13) | 9 | 3 (33) |
| Bone | 3 | 1 (33) | 4 | 2 (50) |

**Table 4** Pretreatment peripheral blood lymphocyte subset analysis determined by flow cytometry

| | BCDT + IL-2 + IFN ($n$ = 23) | BCDT alone ($n$ = 17) | Significance |
|---|---|---|---|
| CD56+ (NK cells) | 238 (± 37) | 212 (± 45) | NS |
| CD3+/HLADR+ (activated T cells) | 134 (± 27) | 113 (± 15) | NS |
| CD4+ (T helper cells) | 771 (± 50) | 756 (± 47) | NS |
| CD8+ (T cytotoxic cells) | 558 (± 54) | 451 (± 69) | NS |

Values are expressed as cells $\mu l^{-1}$ (± s.e.m.), and non-parametric comparisons were made between the two groups using the Mann–Whitney test.

We assessed whether the 3-day subcutaneous IL-2 schedule used in this study before chemotherapy was associated with immune activation. Before any treatment, patients in both arms of the study ($n$ = 23, BCDT/IL-2/IFNα and $n$ = 17, BCDT alone) had similar numbers of peripheral blood lymphocyte subsets (Table 4).

Three days of subcutaneous IL-2 resulted in a significant suppression of the total lymphocyte count in 14 patients randomized to receive IL-2 before chemotherapy: 1.8 (± 0.2, standard error of the mean) × $10^9$ $l^{-1}$ before IL-2, falling to 0.8 (± 0.1) × $10^9$ $l^{-1}$ after IL-2, $P$ = 0.001 (Wilcoxon signed-rank test). Despite the significant fall in the peripheral lymphocyte count induced by IL-2, there was a slight but significant rise in the percentage of NK cells and activated T-cell subsets (Table 5). The percentage of NK cells increased to 19.8 ± 2.8% after IL-2 from 13.5 ± 2.1% before IL-2 ($P$ = 0.048, Wilcoxon signed-rank test), and the percentage of activated T cells rose to 13.6 ± 1.5% after IL-2 from 8.1 ± 0.9% before IL-2 ($P$ = 0.004). Overall, the consequence of this shift in percentage distribution was a lack of any change in the total cell count for these subsets despite the overall lymphopenia (Table 5). There was no change in the percentage of PBLs of the CD4 and CD8 phenotypes, and these absolute cell counts fell.

There was a marked difference in the pattern of treatment-related toxicity between the BCDT + IL-2 + IFN-α-treated patients compared with those who received BCDT alone (Table 5). Haematological toxicity was generally mild, but with combined biochemotherapy there was a statistically significant higher incidence of grade 3–4 thrombocytopenia, 37% vs 13% ($P$ = 0.03) (Table 6). Severe nausea and vomiting were also more frequent patients treated with biochemotherapy, as was hepatic disturbance (Table 6). Severe fatigue and flu-like symptoms together with breathlessness were also more common in those treated with IL-2 + IFN-α in addition to chemotherapy, although none of these other differences were statistically significant.

© *Cancer Research Campaign 1998* 

**Table 5** Mean pre- and post-IL-2 peripheral blood lymphocyte subset analysis determined by flow cytomery in 14 patients with metastatic malignant melanoma randomized to receive biochemotherapy (BCDT + IL-2/IFN-α)

| | Before IL-2 (day −2) | After IL-2 (day 1) | Significance |
|---|---|---|---|
| Total lymphocyte count (× $10^9$ $l^{-1}$) | 1.8 ± 0.2 | 0.8 ± 0.1 | 0.001 |
| CD56+ (NK cells) | | | |
| Cell count | 237 ± 48 | 204 ± 65 | NS |
| % | 13.5 ± 2.1 | 19.8 ± 2.8 | 0.04 |
| CD3+/HLADR+ (activated T cells) | | | |
| Cell count | 132 ± 19 | 117 ± 18 | NS |
| % | 8.1 ± 0.9 | 13.6 ± 1.5 | 0.004 |
| CD4+ (T helper cells) | | | |
| Cell count | 779 ± 69 | 337 ± 33 | 0.001 |
| % | 46.4 ± 3.0 | 43.9 ± 3.5 | NS |
| CD8+ (T cytotoxic cells) | | | |
| Cell count | 500 ± 70 | 252 ± 54 | 0.001 |
| % | 28.0 ± 2.9 | 27.2 ± 2.9 | NS |

Values (± s.e.m.) are expressed both as absolute cell counts (cells $\mu l^{-1}$) and as the mean percentage of total lymphocyte count, taken 3 days after subcutaneous IL-2 and before BCDT chemotherapy. The significance of the difference before and after IL-2 was analysed using the Wilcoxon signed-rank test.

**Table 6** Common toxicity criteria

| | Grade 3–4 | |
|---|---|---|
| **Toxicities** | **BCDT+ IL-2+IFN** | **BCDT alone** |
| Haematological | | |
| Anaemia | 1 (3)[a] | 5 (17) |
| Neutropenia | 7 (20) | 5 (17) |
| Thrombocytopenia | 13 (37) | 4 (13) |
| Neurological | | |
| Motor weakness | 1 (3) | – (0) |
| Hearing loss | – (0) | 2 (7) |
| Sensory loss | 1 (3) | – (0) |
| Gastrointestinal | | |
| Nausea | 6 (17) | 3 (10) |
| Vomiting | 3 (9) | – (0) |
| Diarrhoea | 1 (3) | – (0) |
| Constipation | 1 (3) | 1 (3) |
| Hepatic disturbance | 3 (9) | – (0) |
| Fatigue | 9 (26) | 4 (13) |
| Flu-like symptoms | 7 (20) | 3 (10) |
| Oedema | 1 (3) | – (0) |
| Cardiovascular | – (0) | 1 (3) |
| Breathlessness | 3 (9) | – (0) |
| Infection | 1 (3) | 1 (3) |
| Weight loss | 1 (3) | – (0) |

[a]Numbers in parentheses are percentages.

### DISCUSSION

There have been very few randomized trials that have investigated whether improved response rates could be achieved by the addition of biological therapy to conventional chemotherapy for patients with metastatic melanoma. In one trial, a survival advantage together with an improved response rate was shown for single-agent dacarbazine (DTIC) combined with subcutaneous interferon alpha 2a (IFN-α) compared with DTIC alone (Falkson et al, 1991), although this was not confirmed subsequently in two larger randomized trials (Thomson et al, 1993; Bajetta et al, 1994). Interleukin 2 (IL-2) enhances the cellular immune response by stimulating natural killer cells and specific cytotoxic T-cell expansion. In phase II trials, the addition of intravenous IL-2 to DTIC gave response rates of 22–26% (Dillman et al, 1990; Flaherty et al, 1990; Stoter et al, 1991). Although these results are marginally better than those obtained with DTIC alone, the potential benefit of IL-2 with single-agent chemotherapy has not been studied prospectively in randomized phase III trials.

Biological therapy with combined IFN-α and IL-2 may result in synergistic interactions, with enhanced antigen presentation due to IFN-α-mediated up-regulation of class 1 MHC molecules and consequent improvement in tumour recognition by IL-2-stimulated cytotoxic T cells. In early non-randomized trials, the combination of both agents as sole therapy for metastatic melanoma appeared to be superior to either agent alone (Rosenberg et al, 1989), although this was not confirmed in a subsequent randomized phase III trial of high-dose intravenous IL-2 with or without IFN-α (Sparano et al, 1993). The addition of biological therapy with both IL-2 and IFN-α to chemotherapy may prove effective because of the lack of cross-resistance, as the mechanisms of resistance are different between these modes of therapy. Intensive regimens consisting of intravenous IL-2, subcutaneous IFN-α and platinum/DTIC-based chemotherapy have been associated with response rates of 53–55% (Richards et al, 1992; Legha et al, 1993). Legha et al (1996) treated 30 patients with cisplatin (20 mg $m^{-2}$ d 2–5), DTIC (800 mg $m^{-2}$ d 1) and vinblastine (1.6 mg $m^{-2}$ d 1–5, CVD), followed on days 6–10 and 17–21 by IL-2 continuous i.v. infusion (9 MU $m^{-2}$ $day^{-1}$) with daily subcutaneous IFN-α (5 MU), repeating after 3 weeks. In a recently reported randomized trial, which compared the sequence of biochemotherapy (i.e. CVD/BIO vs BIO/CVD), they showed a higher response rate with chemotherapy followed by IL-2 (66% vs 40% respectively). In a similar biochemotherapy programme, Richards et al (1992) used

© Cancer Research Campaign 1998

the BCDT chemotherapy regimen with intravenous bolus IL-2 (4.5 MU $m^{-2}$ every 8 h and subcutaneous IFN-α (6 MU $m^{-2}$ $day^{-1}$) on days 4–8 and 17–21, repeating after 3 weeks. Of 74 patients treated, the overall response rate was 55% (CR 15%) with a median length of survival exceeding 15 months. While both these studies imply a high response rate for combination biochemotherapy, to date this has not been examined compared with chemotherapy alone in a prospective randomized clinical trial. This is particularly important in view of the severity of the toxicities reported in these phase II trials, including myelosuppression and constitutional effects, such as fever, fatigue, nausea and vomiting, oedema and hypotension. Furthermore, the correct sequence of chemoimmunotherapy requires clarification, as this has varied considerably in previous reports.

In our randomized trial, we chose the BCDT regimen as the standard chemotherapy regimen, based on the phase II data available at the time, which suggested this to be the most active regimen (DelPrete et al, 1994). In view of the severe toxicities experienced by the addition of intravenous IL-2 to chemotherapy (Richards et al, 1992; Legha et al, 1993; Legha et al, 1996), we elected for a subcutaneous delivery for IL-2. The reduced toxicity and improved tolerability of low-dose subcutaneous IL-2 is well documented (Castello et al, 1993) and has been shown previously to induce clear changes in immunological parameters with a significant rise in NK cells (Azpodien et al, 1993). In our study, the IL-2 was administered in a priming dose 3 days before chemotherapy with a view to mobilizing cytotoxic T cells to interact with chemotherapy-damaged tumour cells. Similarly, the IFN-α was administered concurrently with the 3 days of chemotherapy to maximize the activation of the immune response during chemotherapy. Biological therapy with IL-2 before chemotherapy has been administered previously in a series of 27 patients with a reported response rate of 37%, including a 12% CR rate (Demchak et al, 1991). In addition, previous investigators have administered infusional IL-2 before DTIC (Stoter et al, 1991). However, the combination of 3 days subcutaneous moderate-dose IL-2 before chemotherapy followed by 3 days of interferon has not been used in patients with melanoma before. Our study was set up before the results of the recent randomized study reporting a superior response rate for chemotherapy followed by biotherapy (Legha et al, 1996).

Our study failed to show any benefit for the addition of subcutaneous IL-2 and IFN-α to combination chemotherapy in terms of response rate, progression-free survival or overall survival. Two groups were well balanced for known prognostic factors including age, sex, number and distribution of disease sites, performance status and prior therapy. Both groups received a similar number of cycles of treatment (median three courses in each group). For the chemotherapy-alone group, the objective response rate of 27% appears somewhat lower than the 50% response rate reported in previous phase II studies of BCDT (DelPrete et al, 1994). Response rates are frequently lower in randomized trials compared with early phase II data, and of note a similar response rate of 30% was reported recently for BCDT in the Canadian randomized trial of this regimen with/without tamoxifen (Rusthoven et al, 1996). Very few CRs were reported in that trial (< 5%), and none were detected in our chemotherapy-alone group.

There was an increase in some grade 3/4 toxicity in patients who received combined biochemotherapy with BCDT and IL-2/IFN-α, with, in particular, a significantly higher incidence of thrombocytopenia (37% vs 13%). The most obvious non-haematological toxicities included fatigue, flu-like symptoms of fever and chills, and nausea and vomiting. These toxicities are well recognized in patients receiving IL-2 and IFN-α therapy. However, in contrast to the studies of high-dose intravenous IL-2 and subcutaneous IFN-α with chemotherapy (Richards et al, 1992; Legha et al, 1993; Legha et al, 1996), the severity and incidence of these toxicities was lower in our study.

The importance of dose, route of delivery and schedule for biological therapy remains to be determined. In the adjuvant setting, a clear survival benefit has been reported for high-dose IFN delivered initially at a dose of 20 MU $m^{-2}$ intravenously every 5 days for 4 weeks, followed by 10 MU $m^{-2}$ subcutaneously three times a week for 48 weeks (Kirkwood et al, 1996). Whether the lack of benefit in previous adjuvant studies was related to lower doses of IFN or mode of administration (s.c.) is unclear. The high response rates in phase II studies of biochemotherapy that use intravenous doses of IL-2 are badly tolerated by patients of poor performance status and bulk tumour burden, and it remains to be seen in a randomized trial whether the benefit of these intensive regimens is real or not. It is possible that, as with renal cell carcinoma, only patients with a minimal tumour will benefit from strategies that include immunotherapy. A recent randomized trial of intravenous IL-2/IFN-α (s.c.) with/without cisplatin demonstrated higher efficacy for combined biochemotherapy (response rate 36% vs 15%, $P = 0.01$), but failed to show any improvement in progression-free or overall survival (Keilholtz et al, 1996). If dose is important, then it could be argued that we failed to deliver sufficient biological therapy by the subcutaneous route to have any synergistic effect. This appears unlikely because of evidence of an immunological effect in patients treated with biochemotherapy manifest as overall lymphopenia with a relative increase in the percentage of CD56 NK cells and CD3+/HLADR+ activated T cells (Table 5), an effect that was similar to that observed from our previous studies of intra-arterial IL-2 in head and neck cancer (Dadian et al, 1993). It remains unclear whether the changes in overall and subset lymphocyte counts represent a compartment phenomenon (subset analysis after IL-2 was finished was not performed), or whether these changes are clinically relevant in terms of an immunological response against the tumour. However, the enhanced toxicity of biochemotherapy compared with chemotherapy alone would indicate that sufficient biotherapy was being administered to have a systemic effect. Furthermore, there are published data suggesting that low-dose IL-2 may be more effective in vivo than higher doses in selectively activating the $CD56^{bright}$ NK subset, which contains high-affinity IL-2 receptors, without activation of monocytes or lymphocytes (Caligiuri et al, 1993).

It remains to be determined whether biochemotherapy is more effective than chemotherapy alone in the management of patients with metastatic melanoma. As we initially considered that a 20% improvement in response rate would be necessary to counterbalance any additional toxicity due to the addition of biological therapy, we concluded from this randomized phase II study in which the difference in response rates was 4% (95% CI – 17–25%) that a larger phase III study using this regimen was not indicated. Thus, despite a tolerable IL-2/IFN-α biological therapy regimen that resulted in immune activation, little benefit is obtained by including this with conventional BCDT chemotherapy, at the cost of significantly enhanced patient toxicity.

© Cancer Research Campaign 1998

### REFERENCES

Atzpodien J, Korfer A, Franks CR, Poliwoda H and Kirchner H (1990) Home therapy with recombinant interleukin-2 and interferon-α2b in advanced human malignancies. *Lancet* **335**: 1509–1512

Atzpodien J, Kirchner H, Korfer A, Hadman M, Schomburg A, Menzel T, Deckert M, Franzke A, Volkenandt M, Dallmann I, Grosse J and Poliwoda H (1993) Expansion of peripheral blood natural killer cells correlates with clinical outcome in cancer patients receiving recombinant subcutaneous interleukin-2 and interferon-alpha-2. *Tumour Biol* **14**: 354

Atzpodien J, Hanninen EL, Kirchner H, Bodenstein H, Pfreundschuh M, Rebmann U, Metzner B, Illiger HJ, Jaske G, Niesel T, Scholz HJ, Wilhelm S, Pielmeier T, Zakrzewski G, Blum G, Beier J, Muller GW, Duensing S, Anton P, Allhoff E, Jonas U and Poliwada H (1995) Multi-institutional home-therapy trial of recombinant human interleukin-2 and interferon alfa-2 in progressive metastatic renal cell carcinoma. *J Clin Oncol* **13**: 497–501

Bajetta E, Di-Leo A, Zampino MG, Sertoli MR, Comella G, Barduagni M, Giannotti B, Queirolo P, Tribbia G, Bernengo MG, Menichetti ET, Palmeri S, Russo A, Cristofolini M, Erbazzi A, Fowst C, Criscuolo D, Bufalino R and Zilembo N (1994) Multicenter randomized trial of dacarbazine alone or in combination with two different doses and schedules of interferon alfa-2a in the treatment of advanced melanoma. *J Clin Oncol* **12**: 806

Buter J, Sleijfer DT, Winette TA, De Vries EGE, Willemse PHB and Mulder NH (1993) A progress report on the outpatient treatment of patients with advanced renal cell carcinoma using subcutaneous recombinant interleukin-2. *Semin Oncol* **20**: 16–21

Caliguri MA (1993) Low-dose recombinant interleukin-2 therapy; rationale and potential clinical applications. *Semin Oncol* **20** (suppl 9): 3–10

Castello G, Comella P, Manzo T, Napolitano M, Parziale AP, Galati MG, Daponte A, Casaretti R, Celentano E and Comella G (1993) Immunological or clinical effects of intramuscular rIFN alpha-2a and low dose subcutaneous rIL-2 in patients with advanced malignant melanoma. *Melanoma Res* **3**: 43

Dadian G, Riches PG, Henderson DC, Taylor A, Moore J, Atkinson H and Gore ME (1993) Immune changes in peripheral blood resulting from locally directed interleukin-2 therapy in squamous cell carcinoma of the head and neck. *Oral Oncol Eur J Cancer* **29b**: 29–34

DelPrete SA, Maurer LH, O'Donnell J, Forcler RJ and LeMarbre P (1994) Combination chemotherapy with cisplatin, carmustine, dacarbazine, and tamoxifen in metastatic melanoma. *Cancer Treat Rep* **68**: 1403–1405

Demchak PA, Mier JW, Robert NJ, O'Brien K, Gould JA and Atkins MB (1991) Interleukin-2 and high-dose cisplatin in patients with metastatic melanoma: a pilot study. *J Clin Oncol* **9**: 1821–1830

Dillman RO, Oldham RK, Barth NM, Birch R, Arnold J and West WH (1990) Recombinant interleukin-2 and adoptive immunotherapy alternated with dacarbazine therapy in melanoma: a national biotherapy study group trial. *J Natl Cancer Inst* **82**: 1345

Falkson CI, Falkson G and Falkson HC (1991) Improved results with the addition of interferon alpha-2b to dacarbazine in the treatment of patients with metastatic melanoma. *J Clin Oncol* **9**: 1403

Flaherty LE, Redman BG, Chabot GG, Martino S, Gualdoni SM and Heilbrun LK (1990) A phase I–II study of dacarbazine in combination with outpatient interleukin-2 in metastatic malignant melanoma. *Cancer* **65**: 2471–2477

Flaherty LE, Robinson W, Redman BG, Gonzalez R, Martino S, Kraut M, Valdivieso M and Rudolph AR (1993) A phase II study of dacarbazine and cisplatin in combination with outpatient administered interleukin-2 in metastatic malignant melanoma. *Cancer* **71**: 3520

Keilholtz U, Goey FH, Punt CJA, Proebstle T, Salzmann R, Schadendorf D, Lienard D, Scheibenbogen C and Eggermont AMM (1996) A randomised trial of IFNα/IL-2 with and without cisplatin in advanced melanoma: EORTC melanoma cooperative group trial (abstract 1353). *Proc Am Soc Clin Oncol* **15**: 436

Kirkwood JM, Strawderman MH, Ernstoff MS, Smith TJ, Borden EC and Blum RH (1996) Interferon alfa-2b adjuvant therapy of high-risk resected cutaneous melanoma: The Eastern Cooperative Oncology Group Trial EST 1684. *J Clin Oncol* **14**: 7–17

Lakhani S, Selby P, Bliss JM, Perren TJ, Gore ME and McElwain TJ (1990) Chemotherapy for malignant melanomas: combination and high doses produce more response without survival benefit. *Br J Cancer* **61**: 330–334

Legha SS and Buzaid AC (1993) Role of recombinant interleukin-2 in combination with interferon-alfa and chemotherapy in the treatment of advanced melanoma. *Semin Oncol* **20** (suppl. 9): 27–32

Legha SS, Ring S, Bedikian A, Plager C, Eton O, Buzaid AC and Papadopoulos N (1996) Treatment of metastatic melanoma with combined chemotherapy containing cisplatin, vinblastine and dacarbazine (CVD) and biotherapy using interleukin-2 and interferon alpha. *Ann Oncol* **7**: 827–835

Mastrangelo MJ, Nathan F, Faguire HC, et al (1993) Trials with combination chemotherapy and active specific immunotherapy. *Melanoma Res* **3**: 33

Richards JM, Mehta N, Ramming K, et al (1992) Sequential chemoimmunotherapy in the treatment of metastatic melanoma. *J Clin Oncol* **10**: 1338–1343

Rosenberg SA, Lotze MT, Yang JC, Lineham WM, Seipp C, Calabro S, Karp SE, Sherry RM, Steinberg S and White DE (1989) Combination therapy with interleukin-2 and alpha-interferon for the treatment of patients with advanced malignant melanoma. *J Clin Oncol* **7**: 1863–1874

Rosenberg SA, Lotze MT, Yang JC, Topalian SL, Chang AE, Schwartzentruber DJ, Aebersold P, Leitman S, Lineham WM, Seipp CA, White DE and Steinberg SM (1993) Prospective randomised trial of high-dose interleukin-2 alone or in conjunction with lymphokine-activated killer cells for the treatment of patients with advanced cancer. *J Natl Cancer Inst* 622–632

Rumpke PH (1984) The use of chemotherapy in the management of patients with malignant melanoma. *Clin Oncol* **3**: 555–570

Rusthoven JJ, Quirt IC, Iscoe NA, McCulloch PB, James KW, Lohman RC, Jensen J, Burdette-Radoux S, Bodurtha AJ, Silver HKB, Verma S, Armitage GR, Zee B and Bennet K (1996) Randomized, double-blind, placebo-controlled trial comparing the response rates of carmustine, dacarbazine, and cisplatin with and without tamoxifen in patients with metastatic melanoma. *J Clin Oncol* **14**: 2083–2090

Sparano JA, Fisher RI, Sunderland M, Margolin K, Ernest ML, Sznol M, Atkins MB, Dutcher JP, Micetich KC, Weiss GR, Doroshow JH, Aronson FR, Rubinstein LV and Mier JW (1993) Randomised phase III trial of treatment with high-dose interleukin-2 either alone or in combination with interferon alpha-2a in patients with advanced melanoma. *J Clin Oncol* **11**: 1969

Stoter G, Aamdal S, Rodenhuis S, Cleton FJ, Iacobelli S, Franks CR, Oskam R and Shiloni E (1991) Sequential administration of recombinant human interleukin-2 and dacrabazine in metastatic melanoma; a multicenter phase II study. *J Clin Oncol* **9**: 1687–1691

Thomson DB, Adena M, McLeod GR, Hersey P, Gill PG, Coates AS, Olver IN, Kefford RF, Lowenthal RM, Beadle GF, Walpole ET, Boland K and Kingston D (1993) Interferon-alpha 2a does not improve response or survival when combined with dacarbazine in metastatic malignant melanoma: results of a multi-institutional Australian randomized trial. *Melanoma Res* **3**: 133

© Cancer Research Campaign 1998